# A Wideband True Time Delay Circuit Using 0.25 µm GaN HEMT Technology

**DOI:** 10.3390/s23156827

**Published:** 2023-07-31

**Authors:** Jeong-Geun Kim, Donghyun Baek

**Affiliations:** 1 Department of Electronic Engineering, Kwangwoon University, Seoul 01897, Republic of Korea; junggun@kw.ac.kr; 2 School of Electrical Engineering, Chung-Ang University, Seoul 06974, Republic of Korea

**Keywords:** true time delay, GaN, phased array antenna

## Abstract

This paper presents a wideband 4-bit true time delay IC using a 0.25 μm GaN HEMT (High-Electron-Mobility Transistor) process for the beam-squint-free phased array antennas. The true time delay IC is implemented with a switched path circuit topology using DPDT (Double Pole Double Throw) with no shunt transistor in the inter-stages to improve the bandwidth and SPDT (Single Pole Single Throw) switches at the input and the output ports. The delay lines are implemented with CLC π-networks with the lumped element to ensure a compact chip size. A negative voltage generator and an SPI controller are implemented in the PCB (Printed Circuit Board) due to the lack of digital control logic in GaN technology. A maximum time delay of ~182 ps with a time delay resolution of 10.5 ps is achieved at DC–6 GHz. The RMS (Root Mean Square) time delay and amplitude error are <5 ps and <0.6 dB, respectively. The measured insertion loss is <6.8 dB and the input and output return losses are >10 dB at DC–6 GHz. The current consumption is nearly zero with a 3.3 V supply. The chip size including pads is 2.45 × 1.75 mm^2^. To the authors’ knowledge, this is the first demonstration of a true time delay IC using GaN HEMT technology.

## 1. Introduction

**Figure 1:**
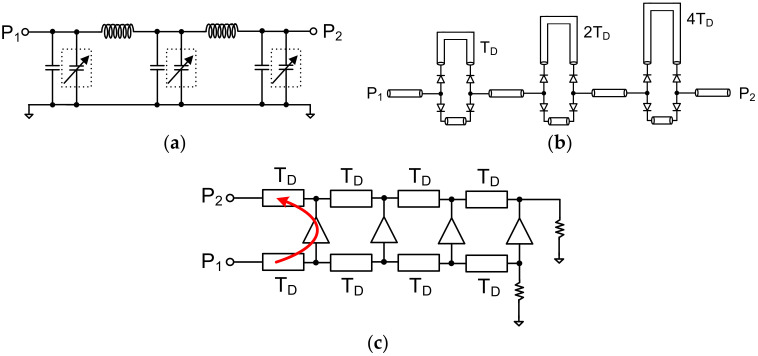
Various true time delay circuit topologies: (**a**) transmission delay line with variable capacitance, (**b**) switched delay line, (**c**) trombone true time delay.

Recently, wide bandgap transistors such GaN or SiC technologies are attracting attention for wideband and high-power applications due to their high breakdown voltage. GaN-on-SiC HEMT technology is popular due to its good thermal properties. There are some papers published on GaN-based amplitude and phase control circuits, such as one detailing a phase shifter and an attenuator for high-power and wideband applications [1]. Phased array antennas using the CMOS process are attracting great interest because CMOS technology can enable low size, weight, power, and cost (SWaP-C) phased array antennas for wireless communication applications [2,3,4,5]. However, the power handling capability is limited in CMOS technology due to the lower inherent breakdown voltage. Phased array antennas with a narrow bandwidth are usually used due to mostly narrowband applications. To construct a narrowband phased array antenna, a phase shifter or a switching phase from the multi-phased local oscillator is widely used. The constant phase characteristic over the frequency in the phase shifter results in a different steered antenna beam position versus the frequency, which is known as the beam squint phenomenon [6,7]. This results in a limited operation frequency bandwidth. However, a true time delay circuit providing a constant time delay over the frequency has become one of the most essential elements in wideband phased array antennas, since a true time delay can prevent beam squinting. Implementing a true time delay circuit as an integrated circuit is difficult because it requires a large chip area to realize the required time delay. Therefore, reducing the chip size is a very important design issue in true time delay design. Figure 1 shows the various true time delay circuit topologies. A true time delay can be implemented with artificial transmission delay lines with variable capacitors, as shown in Figure 1a [8]. However, the characteristic impedance is changed when the capacitance is varied, resulting in a high variation in the return losses depending on the capacitances of the varactors. Most true time delays have been realized using an SPDT switch and an artificial time delay with GaAs or MEMS (Micro Electromechanical System) switches, as shown in Figure 1b [9,10,11,12,13]. Since a conventional true time delay requires many SPDT switches and inductors, which results in a high insertion loss and large chip size, the number of switches and the size of inductors should be reduced. Trombone or active distributed true time delay circuits in Figure 1c have been proposed to improve the insertion loss of the true time delay; however, they cannot provide a compact size or flat time delay performance [14,15,16]. To reduce the delay circuit size, an RC-only tunable delay line circuit has been implemented using CMOS technology [17]. However, it cannot be applied to microwave beamforming applications due to the high insertion loss at high frequencies.

In this paper, a proposed 4-bit true time delay for wideband phased array antennas is presented using 0.25 µm GaN HEMT technology.

## 2. Design of the GaN 4-bit True Time Delay

**Figure 2:**
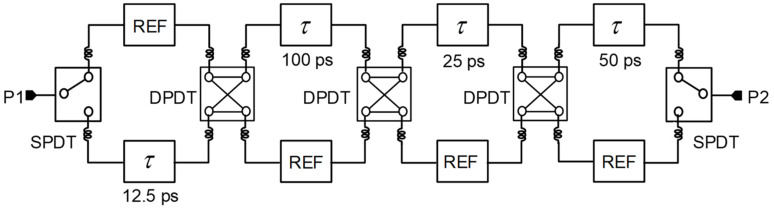
Schematic of the proposed wideband 4-bit GaN true time delay circuit.

**Figure 3:**
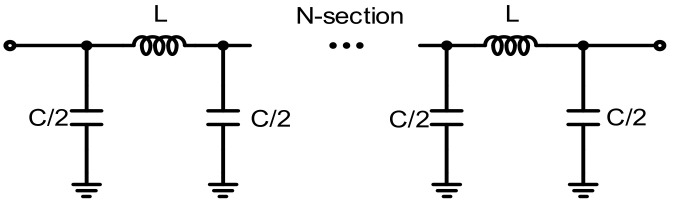
Schematic of the N-section time delay line with a lumped CLC π-network unit cell.

Figure 2 shows the proposed GaN-based 4-bit true time delay IC topology. The proposed 4-bit GaN true time delay circuit is composed of two SPDT switches at the input and output ports, three DPDT switches for the through or cross-path connections, and true time delay elements of 12.5, 25, 50, and 100 ps with LC lumped elements. Switch transistors are generally large in GaN technology; thus, the operating frequency bandwidth is limited due to the large parasitic capacitances. However, even though large switch transistors are used, the parasitic elements of the switch transistors can be nearly identical in each time delay state due to the switched path topology. This results in minimal time delay variations regardless of the time delay states. Therefore, a wide operating bandwidth can be achieved in this topology. The maximum time delay of the designed true time delay IC is 187.5 ps, with a time delay resolution of 12.5 ps. This is equivalent to 56.2 mm in electrical length in air. There are various ways to implement delay element circuits. In this design, a CLC π-network is used as shown in Figure 3. The time delay of the π-network is approximately written as:

(1) $$T_{D}=n\sqrt{\mathrm{LC}}\;$$

where n is the number of sections.

**Figure 4:**
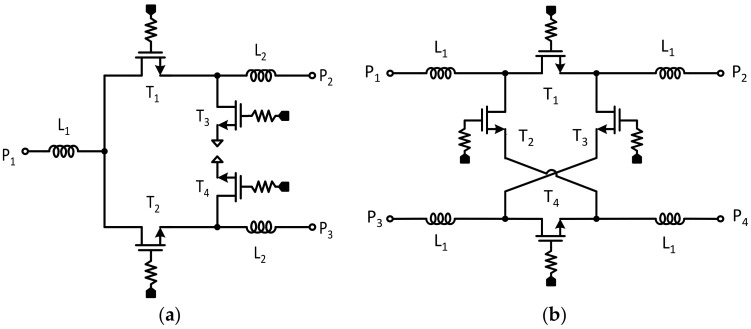
Schematic of (**a**) the GaN SPDT and (**b**) the GaN DPDT switch.

The calculated inductance of L and the capacitance of C are 338 pH and 112 fF, respectively, which obtains the required time delay of 12.5 ps with two sections of the CLC π-network, while maintaining the characteristic impedance of 50 Ω. Four, eight, and sixteen sections of the CLC π-network are used in 25 ps, 50 ps, and 100 ps, respectively. The proposed true time delay employs DPDT switches, which can reduce the number of series switching transistors in the RF signal path. Since the insertion loss of the true time delay is mainly determined by the insertion loss of the series transistors of the SPDT and DPDT switches, the number of series transistors in the RF signal path should be reduced to improve the insertion loss of the true time delay circuit. In the proposed 4-bit true time delay configuration, the RF signal passes through only five series transistors. The insertion loss can be saved by the three series transistors when it is compared to true time delay circuits implemented only with conventional SPDT switches. The proposed true time delay circuit also provides bi-directional operation since it is implemented with only passive devices. As shown in Figure 4a, the series transistors T1 and T2 are used in the SPDT switch for switching the time delay states, and the shunt transistors T3 and T4 are included to improve the RF signal isolation characteristic. For the DPDT switch, only four series transistors, T1, T2, T3, and T4, are used in the DPDT switch in Figure 4b. A reflective switch with shunt transistors is usually implemented in a DPDT switch to improve the isolation characteristic. However, they are not included in this design to reduce the parasitic shunt capacitances because small-size switch transistors are not available in commercial GaN HEMT technology. Even though isolation transistors are not included, a moderate isolation characteristic can be achieved with only series switches at less than 20 GHz. The series inductors L_1_ and L_2_ are implemented to enhance the input and output return loss performances in the SPDT and DPDT switches. A simulated insertion loss of less than 1.3 dB and an isolation greater than 15 dB at 12 GHz are achieved. Only two interconnecting metal layers are available in the GaN HEMT process; thus, a top–bottom stacked metal line is used to implement the inductors and the microstrip transmission lines to improve the metallic loss in the true time delay line. The shunt capacitances in the true time delay line are implemented with MIM capacitors. Electromagnetic simulation was performed carefully to design all the passive devices such as the inductors and MIM (Metal–Insulator–Metal) capacitors making up the true time delay lines and the RF interconnection lines. 

## 3. Measurement Results

**Figure 5:**
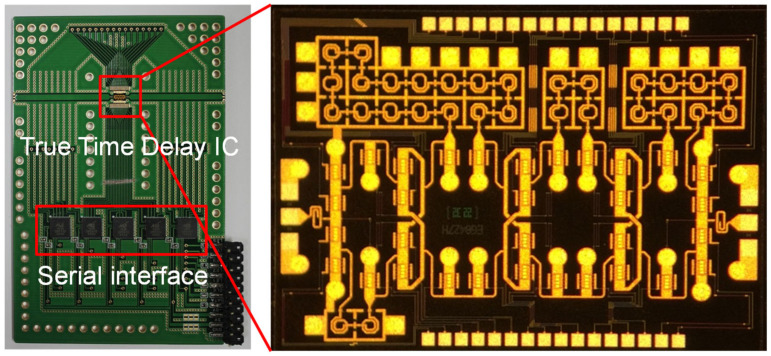
Microphotograph of the GaN-based true time delay circuit and test module.

**Figure 6:**
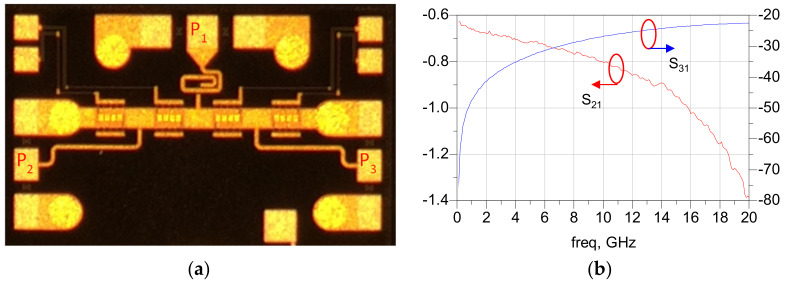
(**a**) Microphotograph of the GaN SPDT switch and (**b**) the measured insertion loss and isolation characteristics.

**Figure 7:**
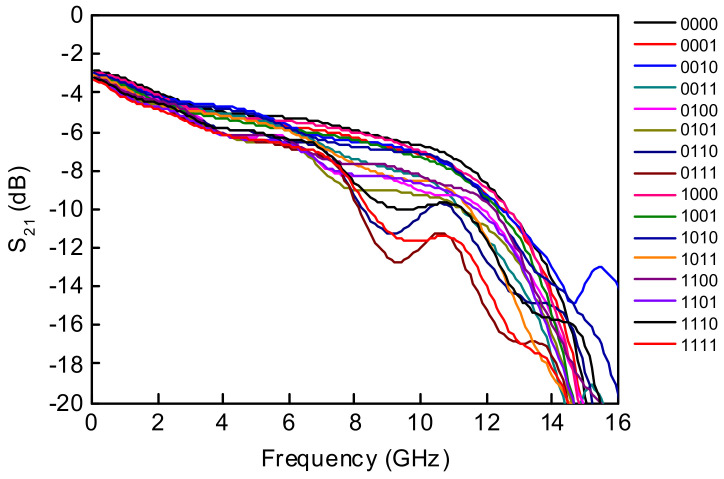
Measured S_21_ of the wideband 4-bit GaN-based true time delay circuit in all sixteen time delay states.

**Figure 8:**
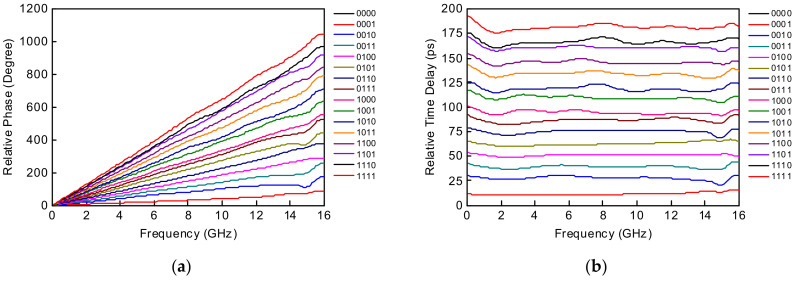
Measured (**a**) relative unwrapped phase and (**b**) time delay characteristic of the GaN-based true time delay circuit in all sixteen time delay states.

The 4-bit wideband true time delay IC was fabricated using 0.25 μm GaN HEMT technology on a GaN-on-SiC substrate. A microphotograph of the 4-bit GaN true time delay IC is shown in Figure 5. The chip size is 2.45 × 1.75 mm^2^, including DC and RF probing pads. The DC and the digital control pads of the GaN true time delay IC are bonded to the test PCB, which includes an external serial-to-parallel interface with a negative gate control voltage generator from the positive 3.3 V voltage to induce a digital control voltage of −3.3 V (off state) and 0 V (on state). Therefore, the complex RF switch control states in the true time delay circuit can be easily configured through the PC-programmable SPI interface. An on-state measurement was performed to characterize the GaN-based true time delay IC using a vector network analyzer and short–open–load–through (SOLT) calibration. Figure 6a shows the test pattern of the GaN SPDT switch. The measured insertion loss (S_21_) of the GaN SPDT switch is less than 1.4 dB and the measured isolation (S_31_) is greater than 20 dB at DC–20 GHz, as shown in Figure 6b, when the series transistor T_1_ and the shunt transistor T_4_ are on and the series transistor T_2_ and the shunt transistor T_3_ are off in Figure 4. Figure 7 shows the measured insertion loss of the GaN true time delay circuit in all sixteen time delay states. An insertion loss of 6.8 dB is achieved in the reference state at DC–6 GHz. The unwrapped phase characteristics and the relative time delay performance of the GaN true time delay IC in all sixteen time delay states are shown in Figure 8. The time delay is calculated from the measured phase of S_21_ as follows:

(2) $$T_{D}=\frac{phase\;of\;S_{21}}{2\pi f}$$

**Figure 9:**
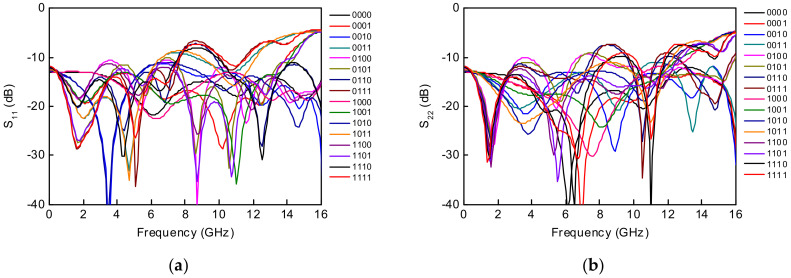
Measured (**a**) S_11_ and (**b**) S_22_ of the wideband 4-bit GaN-based true time delay circuit in all sixteen time delay states.

**Figure 10:**
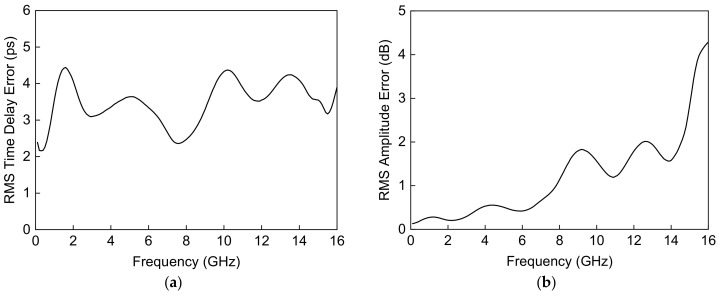
Measured (**a**) RMS time delay error and (**b**) RMS amplitude error of the wideband 4-bit GaN-based true time delay circuit.

A maximum time delay of 182 ps is achieved with a time delay resolution of 10.5 ps at 6 GHz, which is equivalent to 54.6 mm in electrical length in air. There are no overlapping time delay states and there is a flat time delay response at DC–6 GHz. Figure 9 shows the measured input and output return losses in all sixteen time delay states and >10 dB at DC–6 GHz. A measured RMS time delay error of <5 ps and an RMS amplitude error of <0.6 dB are achieved at DC–6 GHz in Figure 10. The total DC power consumption is nearly 0 mW with a 3.3 V supply voltage. The performance comparison with previously published true time delay ICs is summarized in Table 1.

## 4. Conclusions

A wideband 4-bit true time delay IC is presented using 0.25 μm GaN HEMT technology. The proposed true time delay IC is implemented using a DPDT switched path topology with a CLC π-type delay line and exhibits low insertion loss, RMS time delay, and amplitude errors. The maximum time delay range was 182 ps with a time delay resolution of 10.5 ps. The measured insertion loss of the reference state was less than 6.8 dB at DC–6 GHz. The RMS time delay and amplitude errors were less than 5 ps and 0.6 dB, respectively. The DC power consumption was nearly zero. The compact chip size was 2.45 × 1.75 mm^2^, including pads. The proposed GaN-based true time delay IC can be applied to high-power and wideband beam-squint-free phased array radar systems. 

## Figures and Tables

**Table 1:** Performance comparison of the relevant true time delay ICs.

Reference	[5]	[6]	[13]	[16]	This Work
Frequency (GHz)	DC–20	2–20	1–7	6–16	DC–7
Technology	CMOS 0.18 μm	GaAs 0.2 μm	PCB	GaAs 0.25 μm	GaN 0.25 μm
Number of Bits	5	6	7	3	4
Time Delay Range (ps)	106	145	1016	98	182
Time Delay Resolution (ps)	3.3	2.1	8	14	10.5
Insertion Loss of Ref. (dB)	18	11	15.6	3.8–10.5	2.8–8.3
Return Loss of Ref. (dB)	≥12	-	≥12	≥14	≥10
RMS Amplitude Error (dB)	2	-	-	-	≤0.6
RMS Time Delay Error (ps)	1	2.2	-	-	≤5
Chip size (mm^2^)	0.88	5.6	710	1.08 *	4.3

* Core only.

## Data Availability

The data can be obtained from the authors on request.
